# The Sympathetic Nervous System Contributes to the Establishment of Pre-Metastatic Pulmonary Microenvironments

**DOI:** 10.3390/ijms231810652

**Published:** 2022-09-13

**Authors:** Katsuaki Ieguchi, Masabumi Funakoshi, Taishi Mishima, Kohtaro Takizawa, Tsutomu Omori, Fumio Nakamura, Makoto Watanabe, Mayumi Tsuji, Yuji Kiuchi, Shinichi Kobayashi, Takuya Tsunoda, Yoshiro Maru, Satoshi Wada

**Affiliations:** 1 Department of Clinical Diagnostic Oncology, Clinical Research Institute for Clinical Pharmacology and Therapeutics, Showa University, 6-11-11 Kita-karasuyama, Setagaya, Tokyo 157-8577, Japan; 2 Department of Pharmacology, Tokyo Women’s Medical University, 8-1 Kawada-cho, Shinjuku, Tokyo 162-8666, Japan; 3 Clinical Research Institute for Clinical Pharmacology and Therapeutics, Showa University, 6-11-11 Kita-karasuyama, Setagaya, Tokyo 157-8577, Japan; 4 Department of Peripheral Nervous System Research, National Center of Neurology and Psychiatry, National Institute of Neuroscience, 4-1-1 Ogawa-Higashi, Kodaira, Tokyo 187-8551, Japan; 5 Department of Biochemistry, Tokyo Women’s Medical University, 8-1 Kawada-cho, Shinjuku, Tokyo 162-8666, Japan; 6 Department of Pharmacology, Showa University, 1-5-8 Hatanodai, Shinagawa, Tokyo 142-8555, Japan; 7 Pharmacological Research Center, Showa University, 1-5-8 Hatanodai, Shinagawa, Tokyo 142-8555, Japan; 8 Department of Medicine, Division of Medical Oncology, School of Medicine, Showa University, 1-5-8 Hatanodai, Shinagawa, Tokyo 142-8555, Japan

**Keywords:** sympathetic nervous system, metastasis, MDSC, semaphorin, tumor microenvironment, metastatic niche

## Abstract

Emerging evidence suggests that neural activity contributes to tumor initiation and its acquisition of metastatic properties. More specifically, it has been reported that the sympathetic nervous system regulates tumor angiogenesis, tumor growth, and metastasis. The function of the sympathetic nervous system in primary tumors has been gradually elucidated. However, its functions in pre-metastatic environments and/or the preparation of metastatic environments far from the primary sites are still unknown. To investigate the role of the sympathetic nervous system in pre-metastatic environments, we performed chemical sympathectomy using 6-OHDA in mice and observed a decrease in lung metastasis by attenuating the recruitment of myeloid-derived suppressor cells. Furthermore, we note that neuro-immune cell interactions could be observed in tumor-bearing mouse lungs in conjunction with the decreased expression of Sema3A. These data indicate that the sympathetic nervous system contributes to the preparation of pre-metastatic microenvironments in the lungs, which are mediated by neuro-immune cell interactions.

## 1. Introduction

The tumor microenvironment is considered to be a complicated tissue composed of tumor cells, immune cells, vascular endothelial cells, and neurons. Tumor angiogenesis has been intensively investigated and is thought to be a promising therapeutic target, represented by VEGF and integrin αvβ3; on the other hand, neurons in tumor tissue were regarded as non-functional structures until half a century ago [1]. Recent studies have demonstrated that nerve fibers infiltrating into tumor tissues play pivotal roles in tumor growth, angiogenesis, and metastasis [2,3,4,5]. More specifically, it is well-known that psychological behaviors such as depression and chronic psychological stress contribute to tumor formation and progression [6,7]. The sympathetic nervous system (SNS) regulates various physiological functions such as blood pressure and heartbeat in physiological settings [8]. SNS activation via the administration of terbutaline, a specific β2 adrenaline receptor (Adrb2) agonist, causes lymphocytes to stay in lymph nodes and to suppress the immune system [9]. 

Moreover, the SNS has positively contributed to tumor development and progression in prostate cancer and to metastasis to distant organs in breast cancer [10,11]. Eventually, the SNS regulates the recruitment of macrophages and educates tumor-associated macrophages to switch their phenotypes to M2-like macrophages in the tumor microenvironment [11]. Some studies have reported that nerve fibers provide tumor cells that are detached from primary sites with metastatic routes to distant organs and/or tissues [12]. In pathological settings, cancer patients who had been taking a β-blocker, an inhibitor of the β-adrenaline receptors, for more than one year showed prolonged survival compared to patients without β-blocker treatment [13,14,15]. In bone metastasis resulting from breast cancer, the adrenaline receptor contributes to the establishment of metastatic environments mediated by various targets such as osteoclasts [16]. However, the molecular mechanism underlying prolonged survival after β-blocker treatment remains largely unknown. In prostate cancer, sympathetic nerves are often observed in prostate luminal cells. Innervated prostate luminal cells tend to differentiate into neuroendocrine-like cells, which exhibit high proliferative activity and migratory activity in response to Adrb2-mediated stimulation [17]. Sympathectomy during the surgical resection of prostate cancer has been identified as a prognostic factor in a mouse model [10].

We previously reported that myeloid-derived suppressor cells (MDSCs) are recruited to the lungs before the establishment of metastasis and induce lung vascular hyperpermeability mediated by the TLR4-S100A8/A9 axis. S100A8 and S100A9 are inflammatory cytokines that are thought to be endogenous ligands of TLR4 [18,19]. We have also demonstrated that the increased expression of S100A8 in the lungs directly stimulates TLR4 and induces the NF-κB signaling pathway, followed by the increased expression of ephrin-A1 [20]. Stimulation with a soluble form of ephrin-A1 causes cell–cell detachment mediated by the degradation of VE-cadherin [21] and simultaneously upregulates the mRNA expression of *Ccl2*, known as the permeability factor, in vascular endothelial cells [22]. This inflammatory circuit allows circulating tumor cells to easily intravasate into pre-metastatic lungs [23].

Semaphorins are either secreted or membrane-bound proteins that regulate diverse physiological functions such as neural development, and immune functions mediated by their receptors and co-receptors such as plexins and neuropilins [24,25]. Recent studies have demonstrated that semaphorins are expressed in a wide variety of cancers and show multifaceted activity in primary tumors and stroma [26]; their functions in tumor growth, angiogenesis, and metastasis have been intensively investigated [27]. One of the secreted class III semaphorins, termed as Sema3A, is expressed in T cells, monocytes, and macrophages and appears to act as a negative regulator of inflammation [28]. Sema3A is abundantly expressed in endothelial cells in primary tumors and restores normoxic conditions by normalizing vasculature. Sema3A repels endothelial cells elicited by angiogenic factors, such as VEGF-A, in primary tumors and functions as an inhibitor of angiogenesis [29]. Taken together, Sema3A plays a pivotal role in pathological conditions and is a promising target in cancer therapy. However, no molecular-targeting drugs have been clinically used so far.

## 2. Results

### 2.1. Role of the SNS in Tumor Neovascularization

**Figure 1:**
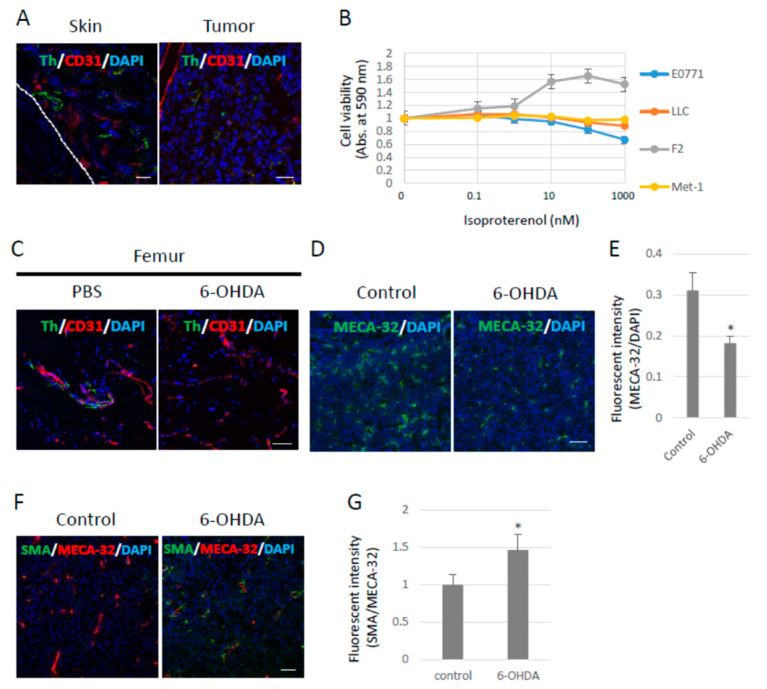
Effects of the sympathetic nervous system on 3LL tumors. (**A**) Sympathetic nerve fibers in primary tumors. Sympathetic nerve fibers labeled with Tyrosine hydroxylase (Th) were found in LLC-derived 3LL tumors and their surroundings. Scale bar: 50 µm. (**B**) Effects of pan-β-agonist (Isoproterenol) on cell proliferation in vitro. LLC, F2, and E0771 cells showed no response to β-agonist stimulation in vitro (*n* = 3). (**C**) Chemical sympathectomy. The femurs were collected from mice administered with 6-OHDA (100 mg/kg) for 14 days and stained with the antibodies anti-Th and anti-CD31 (an endothelial cell marker) antibodies. Sympathetic nerve fibers were significantly reduced in mice treated with 6-OHDA. Scale bar: 10 µm. (**D**) Role of the SNS in angiogenesis. Endothelial cells were visualized with an anti-pan endothelial cell antigen (MECA-32) antibody, and (**E**) tumor angiogenesis was quantified by fluorescence intensity using ZEISS Zen image software. Angiogenesis was decreased in tumors grown in sympathectomized mice (*n* = 5, * *p* < 0.05). Scale bars: 10 µm. (**F**) Role of the SNS in pericyte coverage. Pericytes were visualized with an anti-α-SMA antibody (Scale bar: 10 µm), and (**G**) pericyte coverage was quantified by the fluorescence intensity. Pericyte coverage was enhanced in tumors of sympathectomized mice (*n* = 5, * *p* < 0.05). Scale bar: 10 µm.

It has been reported that neurons penetrate tumors and their surroundings, and that neural penetration contributes to tumor growth mediated by the β-adrenergic receptors expressed in tumors and the stroma. Tumor cells were subcutaneously inoculated into B6 wild-type (WT) mice to examine whether sympathetic nerve fibers would penetrate the primary tumors under our experimental conditions. Sympathetic nerve fibers labeled with tyrosine hydroxylase (Th) were observed in Lewis lung cell carcinoma (LLC)-derived, highly metastasized 3LL tumors. Moreover, sympathetic nerve fibers were found in the skin around the tumors (Figure 1A) as well as around other tumors. The LLC cells showed no proliferative response to isoproterenol (Iso), a synthetic agonist of β-adrenergic receptors, in vitro (Figure 1B). Tumor-bearing (TB)-B6 WT mice were chemically sympathectomized through the administration of 6-hydroxydopamine (6-OHDA) in order to investigate the function of the SNS in primary tumors. To examine the efficacy of chemical sympathectomy by 6-OHDA, we observed that in the femur, in which sympathetic nerve fibers were abundant, treatment with 6-OHDA effectively removed the sympathetic nerve fibers (Figure 1C). Tumor angiogenesis was also significantly inhibited (Figure 1D,E), and pericyte coverage in the tumor vasculature was enhanced, as revealed by α-SMA staining in the primary tumors of the sympathectomized TB mice (Figure 1F,G). These data indicate that the SNS contributed to tumor growth and angiogenesis in the mouse model using 3LL cells.

### 2.2. Decreased Lung Metastasis by Sympathectomy

**Figure 2:**
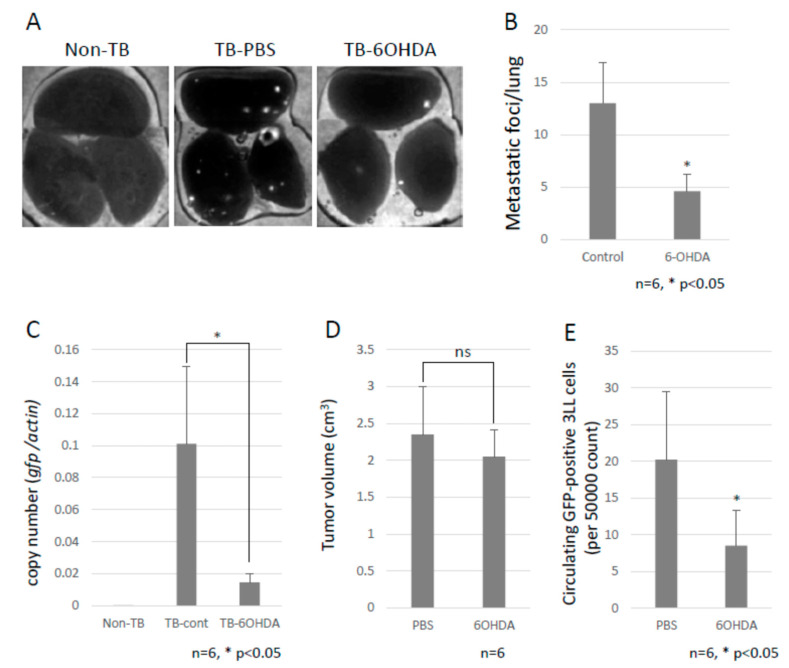
Decreased lung metastasis. (**A**) Effects of chemical sympathectomy on lung metastasis. Metastatic foci were observed with the use of a fluorescence stereoscopic microscope. Representative images are shown. White puncta show the tumor foci in the lungs. (**B**) Lung metastasis. The metastatic foci were counted under a fluorescent stereoscopic microscope (*n* = 5, * *p* < 0.05). (**C**) Lung metastasis was quantified by qPCR. The copy number of *gfp* mRNA in lungs were evaluated by qPCR. Lung metastasis was inhibited by chemical sympathectomy (*n* = 5, * *p* < 0.05). (**D**) Effect of chemical sympathectomy on tumor growth. B6 mice were subcutaneously injected with LLC cells, and the tumor growth was measured with a caliper. Sympathectomy showed no effect on primary tumor growth (*n* = 5). (**E**) Circulating GFP-positive 3LL cells in blood. The number of circulating GFP-positive 3LL cells in the blood of TB mice was analyzed using a flow cytometer. A decreased number of circulating GFP-positive 3LL cells was found in sympathectomized TB mice (*n* = 5, * *p* < 0.05).

To investigate the role of the SNS in metastasis, B6 WT mice were intraperitoneally injected with 6-OHDA to ablate the sympathetic nerve fibers. Subsequently, we tested the effect of the SNS on lung metastasis by using GFP-expressing 3LL cells. Chemical sympathectomy resulted in a marked decrease in lung metastasis (Figure 2A–C and Supplementary Materials Figure S1), but no difference was observed in primary tumor growth (Figure 2D). We also checked the number of circulating GFP-positive 3LL cells in the bloodstream of the mice using a flow cytometer. Circulating GFP-positive 3LL cells were significantly reduced in sympathectomized TB mice (Figure 2E). This result is consistent with the results of the metastasis assay (Figure 2A) and the qPCR analysis of gfp mRNA in the metastatic lungs (Figure 2C). These data suggest that the SNS contributes to the extravasation of tumor cells.

### 2.3. The Establishment of Pre-Metastatic Environments in Lungs by MDSC

**Figure 3:**
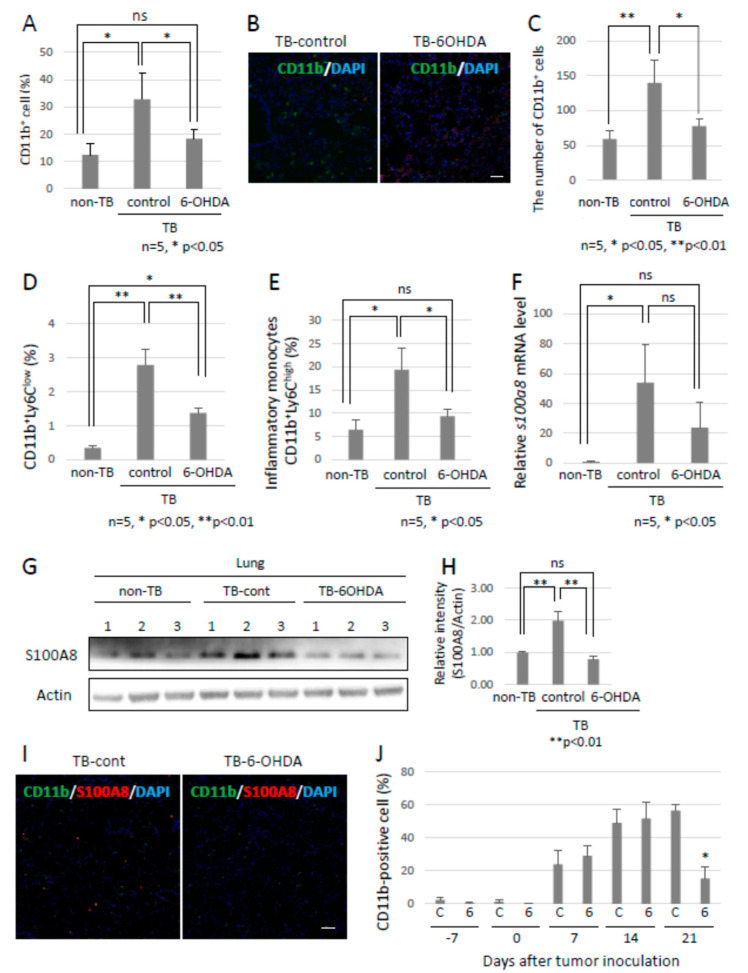
Preparation of pre-metastatic environments in lungs by the SNS. (**A**) Recruitment of CD11b-positive MDSCs into pre-metastatic lungs. Pre-metastatic lungs were analyzed with the use of a flow cytometer and (**B**) immunofluorescent staining (*n* = 5, * *p* < 0.05). Scale bar: 10 µm. (**C**) CD11b-positive cells in pre-metastatic lungs were evaluated using the ZEISS Zen image software. Recruitment of MDSCs to pre-metastatic lungs of sympathectomized TB mice was decreased (*n* = 5, * *p* < 0.05, ** *p* < 0.01). (**D**,**E**) Monocytic MDSCs in pre-metastatic lungs. Recruitment of monocytic MDSCs, defined as CD11b^+^Ly6C^+^, into pre-metastatic lungs. Recruitment of MDSCs was significantly reduced in pre-metastatic lungs of sympathectomized mice (*n* = 5, * *p* < 0.05, ** *p* < 0.01). (F–I) S100A8 expression in pre-metastatic lungs. S100A8 expression levels were tested by (**F**) qPCR (*n* = 5, * *p* < 0.05), (**G**,**H**) immunoblotting, and (**I**) immunofluorescent staining. Band intensity of S100A8 was evaluated with NIH ImageJ software (*n* = 3, ** *p* < 0.01). Fluorescence intensity was estimated using the ZEISS Zen image software. Expression levels of S100A8 were markedly decreased in the pre-metastatic lungs of sympathectomized mice. Scale bar: 10 µm. (**J**) MDSCs in peripheral blood. The number of CD11b-positive cells in the peripheral blood of sympathectomized TB mice was estimated using a flow cytometer; the CD11b-positive cells were markedly reduced in the peripheral blood of sympathectomized TB mice (*n* = 5, * *p* < 0.05).

**Figure 4:**
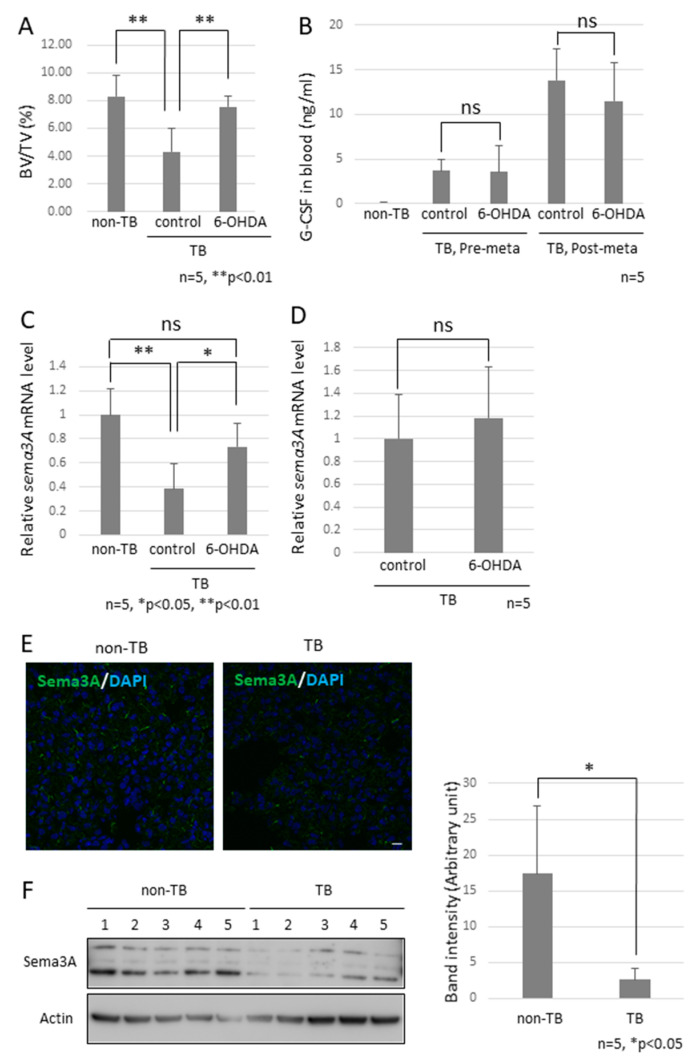

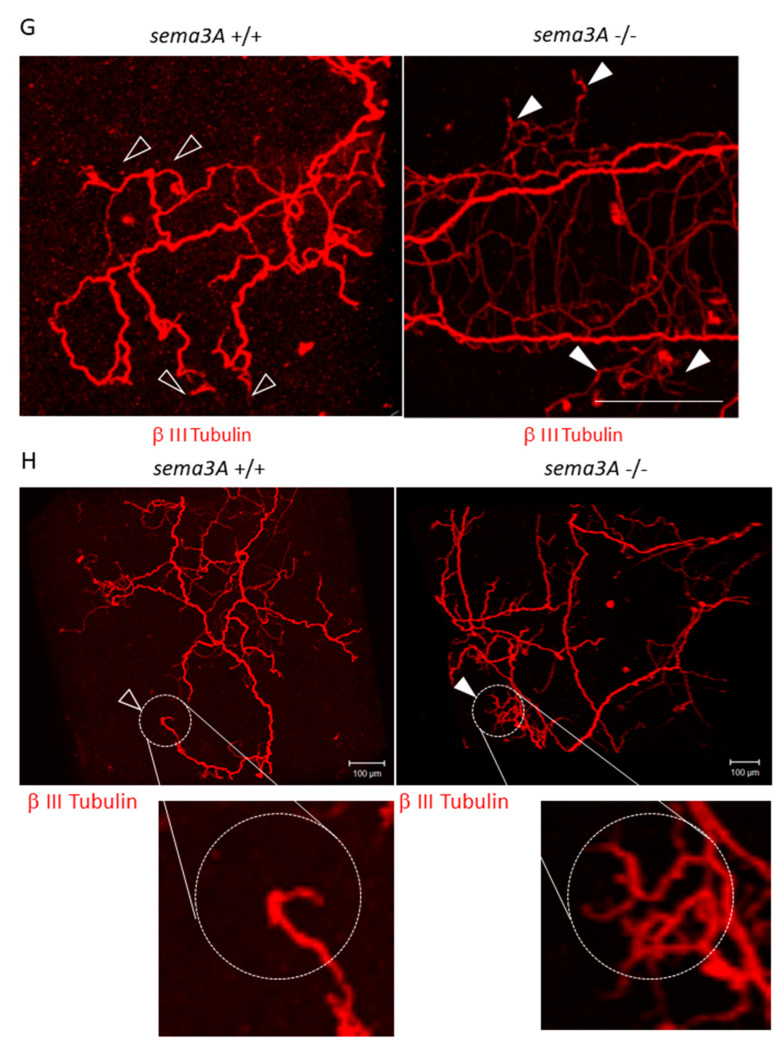
Effects of decreased Sema3A expression on pre-metastatic lungs. (**A**) Bone mass index of TB mice. The femurs of TB mice were collected and stained with hematoxylin and eosin. Bone morphological measurements were performed using the tissue sections. Sympathectomized mice were protected from bone resorption (*n* = 5, ** *p* < 0.01). (**B**) G-CSF levels in serum. G-CSF levels in serum were estimated by ELISA. Serum levels of G-CSF in sympathectomized TB mice were comparable to those in TB control mice (*n* = 5). (**C**) Messenger RNA expression of *Sema3A* in lungs (*n* = 5, * *p* < 0.05, ** *p* < 0.01) and (**D**) bone marrow. Messenger RNA levels of *Sema3A* were determined by qPCR. Expression levels of *Sema3A* were decreased in the lungs of TB mice, but decreased expression was recovered by sympathectomy (*n* = 5). (**E**,**F**) Sema3A expression in lungs. Sema3A expression in the lungs of TB mice was also determined by immunofluorescent staining (Scale Bar: 10 µm) and immunoblotting. The band intensity was quantified by NIH Image J. Sema3A expression levels were attenuated in TB mouse lungs. (**G**,**H**) Role of Sema3A in neural network in lungs. Lungs were treated with CUBIC buffer for cleaning and stained with anti-Tuj1 antibody (βIII Tubulin). The transparent lungs were observed, and the Z-stack images were obtained using a confocal microscope (**G**) and a fluorescent stereoscopic microscope (**H**). Closed arrow heads showed non-integrated neural projections with branching nerve ends. Open arrow heads correspond to location of closed arrow heads in *Sema3A* KO lung. Non-integrated neural projections and branching nerve ends were not observed in WT mouse lungs. Scale Bars: 100 µm.

Since the role of the SNS in metastasis remained to be elucidated, we analyzed the function of the SNS in the establishment of pre-metastatic environments by using pre-metastatic lungs obtained from sympathectomized TB mice. Our previous studies showed that CD11b-positive cells were recruited to the pre-metastatic lungs, and the recruited CD11b-positive cells in the lungs of sympathectomized TB mice were significantly fewer than those in the lungs of TB control mice (Figure 3A) 30. Immunofluorescence staining supported these results (Figure 3B,C). We also analyzed MDSC content in the lungs. The recruitment of monocytic MDSCs, defined as CD11b^+^Ly-6C^low^, and inflammatory monocytes, defined as CD11b^+^Ly-6C^high^, was markedly reduced in the lungs of sympathectomized TB mice (Figure 3D,E), although no difference was found in the granulocytic MDSC population. In control mouse lungs, mRNA and protein levels of S100A8, which is a master positive regulator in the establishment of pre-metastatic environments in mouse lungs, were upregulated, but not in the lungs of sympathectomized TB mice (Figure 3F–I). Moreover, a decreased number of CD11b-positive cells was observed in the peripheral blood of sympathectomized TB mice (Figure 3J). We hypothesized that the decreased number of CD11b-positive cells in the peripheral blood of sympathectomized TB mice was caused by the exhaustion of hematopoietic stem and progenitor cells (HSPC) from the bone marrow. To test this possibility, we performed bone morphological measurements to determine the bone mass index. Contrary to our expectations, sympathectomy markedly prevented bone loss, whereas the TB control mice showed significant bone loss (Figure 4A). It has been reported that granulocyte colony-stimulating factor (G-CSF) stimulation induces HSPC mobilization from the bone niche 31, which resulted in the accumulation of MDSCs in a primary tumor in an experimental model 32 and in a pathological setting 33. Additionally, we checked the serum levels of G-CSF in the TB mice. However, the serum levels of G-CSF in sympathectomized TB mice were comparable to those in the control mice (Figure 4B). Taken together, the decreased number of CD11b-positive cells in the peripheral blood of sympathectomized TB mice may be due to a lower supply of MDSCs from the bone marrow via an unknown mechanism.

### 2.4. Role of Sema3A in Pre-Metastatic Environments in Lungs

To examine the factor of bone loss observed in TB mice, we focused on osteoprotection regulated by a functional balance between osteoblasts and osteoclasts [30] and then checked the mRNA expression levels of *Sema3A* since the knockout mice showed a defect in osteoprotection [31]. We found a marked reduction in *Sema3A* mRNA levels in pre-metastatic lungs, but not in the bone marrow (Figure 4C,D). This downregulation is consistent with our previous microarray data [32]. Subsequently, we analyzed Sema3A expression in pre-metastatic lungs because reduced Sema3A expression results in the loss of repulsive signals for neurite outgrowth and leads to the loss of nerve fiber integrity [33,34]. No difference in the localization of Sema3A was found in the lungs, although Sema3A expression was obviously decreased in the results from immunofluorescence staining and immunoblotting (Figure 4E,F).

### 2.5. Analysis of the Neuro-Immune Cell Interactions during the Establishment of Pre-Metastatic Environments in the Lungs

**Figure 5:**
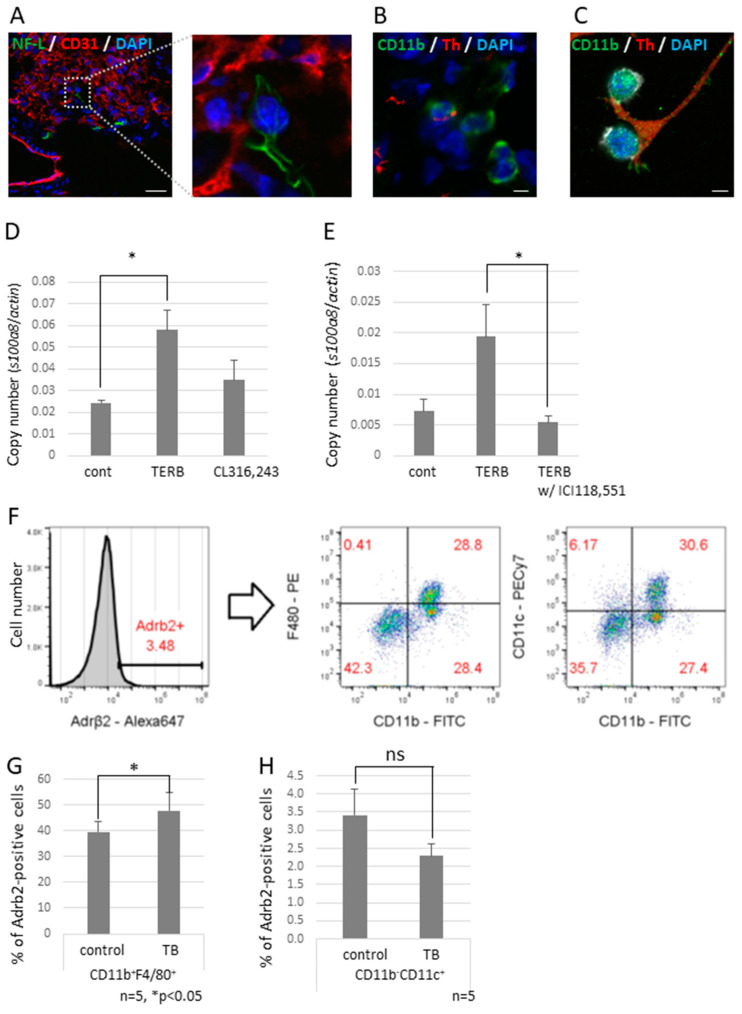
Neuro-immune cell interactions in pre-metastatic lungs. (**A**) Interactions between sympathetic nerve fibers and non-endothelial cells. An NF-L-positive neuron interacted with a non-endothelial cell in pre-metastatic lungs. Enlarged image is the section enclosed by the broken line. Scale Bar: 10 µm. (**B**) Neuro-immune cell interaction. Pre-metastatic lungs were stained with anti-CD11b and anti-Th antibodies and observed using a confocal microscope. A Th-positive neuron interacted with CD11b-positivel cells. Scale Bar: 10 µm. (**C**) Co-culture of neurons and macrophages in vitro. Differentiated PC12 cells were co-cultured with J774.1 cells. Neuro-immune cell interactions were also found in vitro. Scale Bar: 10 µm. (**D**) Induced cytokine expression by the β2 adrenaline receptor agonist. J774.1 cells were stimulated with Terbutaline, a β2 adrenaline receptor selective agonist, or CL316,243, a β3 adrenaline receptor selective agonist; mRNA expression levels of *S100a8* were tested by qPCR (*n* = 3, * *p* < 0.05). (**E**) β2 adrenaline receptor-mediated up-regulation of *S100a8*. Expression of *S100a8* was up-regulated by TERB and abolished by pre-incubation with ICI118,551, a β2 selective antagonist (*n* = 3, * *p* < 0.05). (**F**–**H**) Adrb2 expression in MDSCs. Pre-metastatic lungs were stained with anti-Adrb2, anti-CD11b, anti-CD11c, and anti-F4/80 antibodies and analyzed using a flow cytometer. Adrb2-positive cells were divided into CD11b^+^F4/80^+^ and CD11b^−^CD11c^+^ populations. Both groups expressed Adrb2, and Adrb2 expression was induced in bone marrow-derived macrophages defined as CD11b^+^F4/80^+^, but not in alveolar macrophages defined as CD11b^−^CD11c^+^ (*n* = 5, * *p* < 0.05).

To investigate the effect of decreased expression of Sema3A on neurite outgrowth, we tried to observe neural networks in a piece of the whole lobe of the lung of a *Sema3A* KO mouse. We found that non-integrated nerve fibers ended around the bronchioles and apical pulmonary regions (Figure 4G,H). We also found that some nerve fiber ends interacted with CD11b-positive and CD31-negative cells, which are defined as bone marrow-derived immune cells (Figure 5A,B), but this was rarely observed in control mouse lungs. Neuro-immune cell interactions were also observed in vitro using differentiated PC12, which is a rat pheochromocytoma cell line, along with nerve growth factor (NGF) and J774.1 cells, which is a cultured mouse macrophage cell line. These data suggest that CD11b-positive cells were educated by sympathetic neurons and contributed to preparing the pre-metastatic environments in the lungs. We previously reported that the increased expression of S100A8 in a pre-metastatic lung established metastatic environments mediated by TLR4 [32]. To check if adrenergic receptor-mediated signaling upregulates the expression of *S100a8,* the J774.1 cells were stimulated with terbutaline (Terb), a specific agonist for Adrb2, or with CL316,243, a specific agonist for the β3 adrenaline receptor; this resulted in a significant increase in *S100a8* mRNA levels (Figure 5D) and failed to upregulate the expression with ICI118,551, a specific β2 adrenaline receptor antagonist (Figure 5E). Moreover, we tested whether CD11b-positive cells in mouse lungs expressed Adrb2. Adrb2-positive cells were analyzed by flow cytometry using anti-CD11b, anti-CD11c, and anti-F4/80 antibodies (Figure 5F). Both bone marrow-derived macrophages (CD11b^+^F4/80^+^) and alveolar macrophages (CD11b^−^CD11c^+^) expressed Adrb2, and Adrb2 in bone marrow-derived macrophages was slightly upregulated in the lungs of TB mice, but not in alveolar macrophages (Figure 5G,H).

### 2.6. Inhibition of Lung Metastasis by the β-Blocker Propranolol

**Figure 6:**
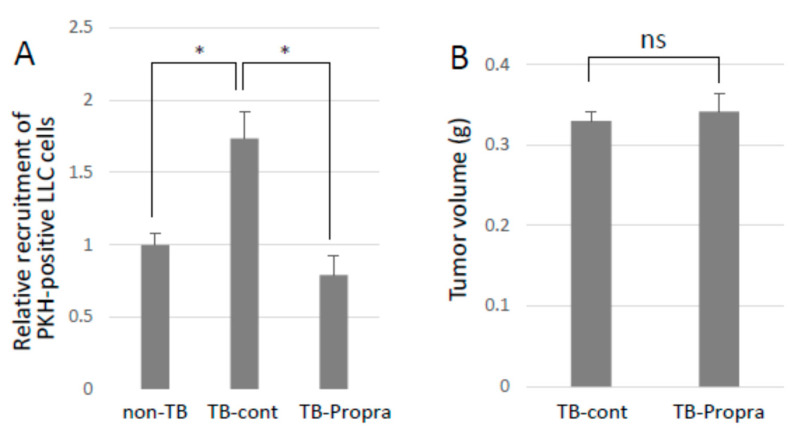
Effects of β-blocker on lung metastasis. (**A**) Recruitment of CTCs to pre-metastatic lung. PKH-labeled LLC cells were injected into the tail veins of TB mice, and the lungs were analyzed by a flow cytometer to detect PKH-positive cells recruited to the lungs. Recruited cells to pre-metastatic lungs were inhibited by β-blocker treatment (*n* = 5, * *p* < 0.05). (**B**) The effect of β-blocker on tumor growth. Primary tumors of mice treated with β-blocker were weighed after being sacrificed. β-blocker treatment showed no effect on tumor growth (*n* = 5).

Breast cancer patients who had been taking β-blockers for more than one year prolonged their survival as compared with those without β-blocker treatment [13,14,15]. Propranolol was intraperitoneally administered to mice from 7 days before to 14 days after tumor inoculation in order to examine whether a β-blocker inhibited lung metastasis. Propranolol treatment successfully inhibited experimental lung metastasis, with no effect on primary tumor growth (Figure 6A,B). However, propranolol administration somehow resulted in an increase in *S100a8* mRNA expression, but had no effect on *Ccl2* mRNA expression, which enhances lung vascular permeability and metastasis. Unexpectedly, spontaneous metastasis was not inhibited by propranolol administration, but metastatic tumor foci in the lungs seemed to be smaller than those in the lungs of TB control mice.

## 3. Discussion

The role of the SNS in primary tumors has been intensively investigated, and it has been reported that the SNS contributes to tumor angiogenesis, tumor growth, and polarization of tumor-associated macrophages [11]. Adrenaline receptors are expressed in many tumors and enhance cell proliferation [35]. In fact, sympathetic nerve fibers were observed in the primary tumors and their stroma under our experimental conditions (Figure 1A). This neural projection into primary tumors has also been observed under other experimental conditions and pathological settings, such as in prostate cancer [16]. Therefore, in this study, we used tumor cell lines that have no proliferative response to β-agonist stimulation in order to investigate the functions of the SNS in pre-metastatic environments. Some meta-analyses that used cohorts with a medication history and the survival rate of cancer patients have demonstrated that β-blocker treatment prolongs survival in breast cancer patients. However, the molecular mechanisms by which β-blockers prolong the survival of cancer patients remain to be fully elucidated. In this study, we first focused on the roles of the SNS in pre-metastatic microenvironments. Bronchial asthma often develops when the parasympathetic nervous function is predominant as compared with the sympathetic nervous function. The balance between sympathetic and parasympathetic nerve functions is important for lung homeostasis. The transient stimulation of the β2 adrenaline receptor with specific agonists, such as salbutamol, reduces the number of T and B cells in peripheral blood [36]. These data indicate that inflammation mediated by the TLR4/S100A8 axis should be attenuated when immune cells are innervated by sympathetic nerves in pre-metastatic environments or the blood. Attenuated inflammation by transient sympathetic innervation in pre-metastatic environments may inhibit metastasis. However, chronic stress in TB mice increases the risk of metastasis without affecting primary tumor growth (9). Moreover, the abolishment of sympathetic nerve activity decreases the recruitment of MDSCs into pre-metastatic environments. These results suggest that transient sympathetic nerve innervation by salbutamol at a non-physiological dosage (100 mg/kg) causes immune cells to stay in the lymph nodes, thus decreasing inflammatory actions; on the other hand, chronic sympathetic innervation by physiological factors, such as psychological stress, increases inflammatory actions, thereby enhancing metastatic risks.

The SNS plays an important role in the regulation of physiological functions such as blood pressure and the immune system [37]. Accordingly, chemical sympathectomy causes physiological changes and may affect anti-tumor immunity by itself. Moreover, reactive oxygen species (ROS) generated by chemical sympathectomy is well-known to induce DNA damage. The chemical ablation of sympathetic nerves may be a limited experimental model with which to examine the role of the SNS in the establishment of a pre-metastatic environment. Further investigations will be required to test the actual function of the SNS in this model. 

We hypothesized that β-blocker treatment would inhibit the establishment of pre-metastatic microenvironments in the lungs. However, it unexpectedly failed to inhibit the mRNA expression of *S100a8*, which is an important factor for the establishment of pre-metastatic environments in the lungs of TB mice, although the inhibitory effect of β-blockers on macrophages had already been observed in vitro (Figure 5E). This difference may be due to the side effects of β-blockers on the lungs since the adrenaline receptors are also expressed in the bronchioles and other CD11b-positive immune cells (Figure 5F). Moreover, treatment with a β-blocker failed to inhibit spontaneous lung metastasis, although the recruitment of tumor cells injected into the tail veins of TB mice was inhibited by treatment with a β-blocker. In the spontaneous metastasis assay with the β-blocker treatment, the number of metastatic foci showed no statistical significance, but metastatic tumor foci appeared to be smaller than those in the control mouse lungs. Therefore, β-blocker treatment might be effective in the re-growth step during the establishment of metastasis in distant organs.

**Figure 7:**
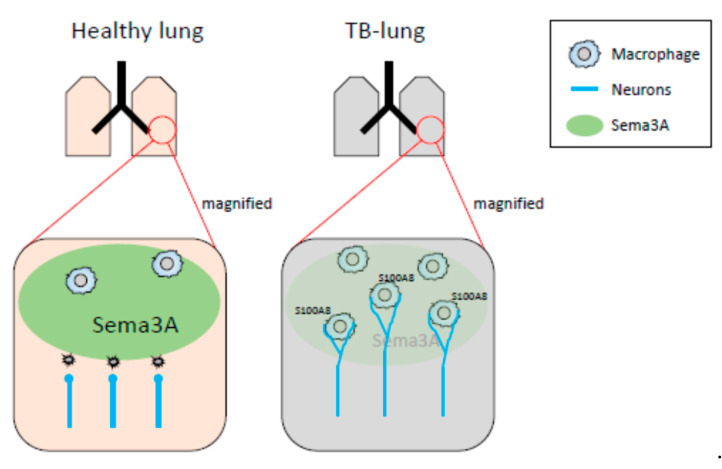
Schematic representation of neuro-immune cell interactions by loss of Sema3A expression. In healthy lungs, Sema3A acts as a guardian and blocks neural invasion and interactions between neurons and macrophages. The decreased expression of Sema3A in pulmonary pre-metastatic environments leads to loss of neural integrity, resulting in an increase in neuro-immune cell interactions. Expression of S100A8 might be up-regulated by the interactions mediated through the adrenaline β2 receptor.

In our previous and present study, decreased mRNA expression of *Sema3A* was observed in pre-metastatic lungs [32]. It has been reported that Sema3A functions as an anti-inflammatory factor. Under physiological conditions, neural projections are supposed to be strictly regulated by Sema3A-mediated repulsive signals, and sema3A may act as a guardian that interrupts neural invasion. When Sema3A expression is decreased in inflammatory regions, precisely regulated neural projections might be dysregulated, while neurons extend their neurites to the inflammatory region (Figure 7). In fact, the neural projections in the lung of the *Sema3a* KO mouse were different from those of WT mice. In particular, branching at the terminal ends of neurons in the lungs of *Sema3a* KO mice was undeniably different from that in WT mouse lungs (Figure 4G,H). Moreover, it has been reported that immune cells, including macrophages, express Nrp1, a receptor of Sema3A, and regulate immune functions [38,39,40]. Consequently, Nrp-1-positive immune cells could be free to move to Sema3A-low areas and be innervated by sympathetic neurons. Further investigations are required to unveil Sema3A-mediated neuro-immune interactions. Moreover, osteoprotection regulated by Sema3A expression was severely inhibited in the femurs of TB mice (Figure 4A). Some studies have demonstrated that the SNS regulates osteoclast differentiation, which directly causes bone disruption [27,41]. Based on the results shown in Figure 4C,D, lung-derived Sema3A may partially function as an osteoprotective agent. Further investigations are needed to examine the role of lung-derived Sema3A in osteoclast differentiation during tumor growth.

Overall, we first reported that sympathetic nerve fiber ends interacted with macrophages and thereby enhanced the expression S100A8, which recruited MDSCs to the pre-metastatic pulmonary environments mediated by the β2 adrenaline receptor in our experimental conditions. However, we have not yet ascertained the occurrence of these observations in other metastatic models. This point is the limitation of our study. Therefore, further investigations are required to demonstrate that neuro-immune interactions in pre-metastatic environments promote metastasis by using other metastatic experimental models and metastatic cell lines.

## 4. Materials and Methods

### 4.1. Materials

Antibody against tyrosine hydroxylase was purchased from Merck Millipore (Cat. No. AB152, Burlington, MI, USA). Alexa647-conjugated anti-Adrb2 antibody was purchased from Bioss (Cat. No. BS-0947R-A647, Woburn, MI, USA). The FITC-conjugated anti-smooth muscle actin antibody was obtained from Sigma-Aldrich (Cat. No. F3777, St. Louis, MO, USA). Anti-Tuj1 and anit-F4/80 antibodies were purchased from BioLegend (Cat. No. PRB-435P and 123114, San Diego, CA, USA). Anti-Neurofilament-L and-actin antibodies were bought from Cell Signaling Technology (Cat. No. 2837, 4970, Danvers, MA, USA). Anti-CD31 and MECA-32 antibodies were purchased from BD Biosciences (Cat. No. 550274 and 550563; Franklin Lakes, NJ, USA). Antibodies against CD11b, Ly-6c, Ly-6G, and CD11c as well as each isotype control were obtained from BD Biosciences (Cat. No. 57397, 560593, 551460, 117346). Anti-Sema3A antibody was purchased from Abcam (Cat. No. ab199475, Cambridge, UK). Isoproterenol, terbutaline, ICI118,551, and CL316,243 were procured from Sigma-Aldrich. Anti-S100A8 antibody was purchased from Santa Cruz Biotechnology (SC-812, Santa Cruz, CA, USA). Hexa-hydroxydopamine hydrochloride was obtained from Sigma-Aldrich St. Louis (St. Louis, MO, USA).

### 4.2. Cell Culture

The 3LL cells were obtained from the Cell Resource Center for Biomedical Research, Institute of Development, Aging and Cancer at Tohoku University, and they were cultivated, as described previously [42]. E0771, a mammary carcinoma cell line, and LLC, the Lewis lung carcinoma cell line, were obtained and cultivated, as previously described [43]. Met-1 cell, which is a mammary carcinoma generated from MMTV-PyMT mice, was kindly provided by Dr. Borowsky and was cultivated, as previously described [44]. F2 cells, which come from a mouse endothelial cell line, were established from UV-induced tumors and then cultivated, as previously described [45]. The 3LL cells were transfected with the pEGFP-N3 empty vector and selected with 400 µg/mL of G418. The 3LL-EGFP-positive cells were isolated by FACS (MoFlo Astorios, Beckman Coulter, Brea, CA, USA). The J774.1 cells were purchased from ATCC, cultivated in DMEM (Fujifilm Wako, Osaka, Japan), and then supplemented with 10% fetal bovine serum and penicillin/streptomycin. 

### 4.3. Quantitative RT-PCR

The J774.1 cells were stimulated with β adrenaline receptor agonists, with or without β adrenaline receptor antagonists. Total RNA was purified using ISOGEN II (Nippon Gene, Tokyo, Japan). Reverse transcription was performed using ReverTra Ace qPCR RT Master Mix along with gDNA Remover according to the manufacturer’s instructions (TOYOBO, Osaka, Japan). qPCR was performed by Step-One plus (Thermo Fisher Scientific, Waltham, CA, USA).

### 4.4. Animal Study

C57BL/6J mice were purchased from CLEA (Tokyo, Japan) and used for the experiments at 8–10 weeks old. Chemical sympathectomy was performed by intraperitoneal injections of 6-OHDA (100 mg/kg) in PBS with 10% vitamin C, three times a week, from 2 weeks before tumor inoculation until sacrifice. B6 mice were subcutaneously inoculated with 2 × 10^5^ 3LL-EGFP cells or 5 × 10^5^ LLC cells and intraperitoneally injected with propranolol every day, three days after tumor inoculation until sacrifice. Tumor-bearing mice were sacrificed 3 weeks after 3LL cell inoculation for the spontaneous metastasis assay and 2 weeks after LLC cell inoculation for pre-metastatic analysis, followed by the analysis of peripheral blood, femurs, lungs, and tumors. The knockout mouse of *Sema3A* was kindly provided by Dr. Yagi [46]. All procedures performed on mice were approved by the Animal Research Committee of Tokyo Women’s Medical University.

### 4.5. Immunofluorescent Staining for Whole Lung Observation

The mice were sacrificed with CO_2_ gas and washed with PBS under physiological pressure. The lungs were placed in 4% paraformaldehyde for 2 h, followed by extensive washing with PBS. Subsequently, the lungs were permeabilized with 1% Triton X-100 (St. Louis, MO, USA) in PBS containing 2% skim milk, followed by overnight incubation with an anti-Tuj1 antibody. The lungs were washed thrice with PBS containing 0.2% Tween 20 for 10 min. The lungs were fixed again with 4% paraformaldehyde for 10 min after Alexa555-conjugated anti-rabbit IgG incubation and then placed in CUBIC-L and CUBIC-R solutions to make the lungs transparent (Tokyo Chemical Industry). The transparent lungs were observed, and Z-stack images were obtained using an LSM710 confocal microscope (Zeiss, Oberkochen, Germany) or a stereoscopic microscope (LEICA Microsystems, Wetzlar, Germany).

### 4.6. Flowcytometric Analysis

The lungs of tumor-bearing (TB) mice were enzymatically digested in DMEM containing 1 µg/mL collagenase, 1 mg/mL dispase, and ribonuclease, and then stained with the appropriate antibodies. The cells were analyzed using CytoFLEX (Beckman Coulter). Raw data were analyzed using the FloJo analysis software (Becton Dickinson, Franklin Lakes, NJ, USA).

### 4.7. Statistical Analysis

The data are expressed as mean ± s.d. or SEM. Comparisons between the two groups were performed using a two-tailed paired Student’s *t*-test. In all experiments, statistical significance was set at *p* < 0.05. 

## Data Availability

Data is contained within the article or supplementary material.

## Supplementary Materials

The following supporting information can be downloaded at: https://www.mdpi.com/article/10.3390/ijms231810652/s1.
