# Supplementary material for: The Sympathetic Nervous System Contributes to the Establishment of Pre-Metastatic Pulmonary Microenvironments
Source: Int J Mol Sci. 2022 Sep 13;23(18):10652. doi: 10.3390/ijms231810652 (PMC9501257; doi:10.3390/ijms231810652)
Supplement: Supplementary file 1 [file ijms-23-10652-s001.zip › ijms-1899993-supplementary.pdf]

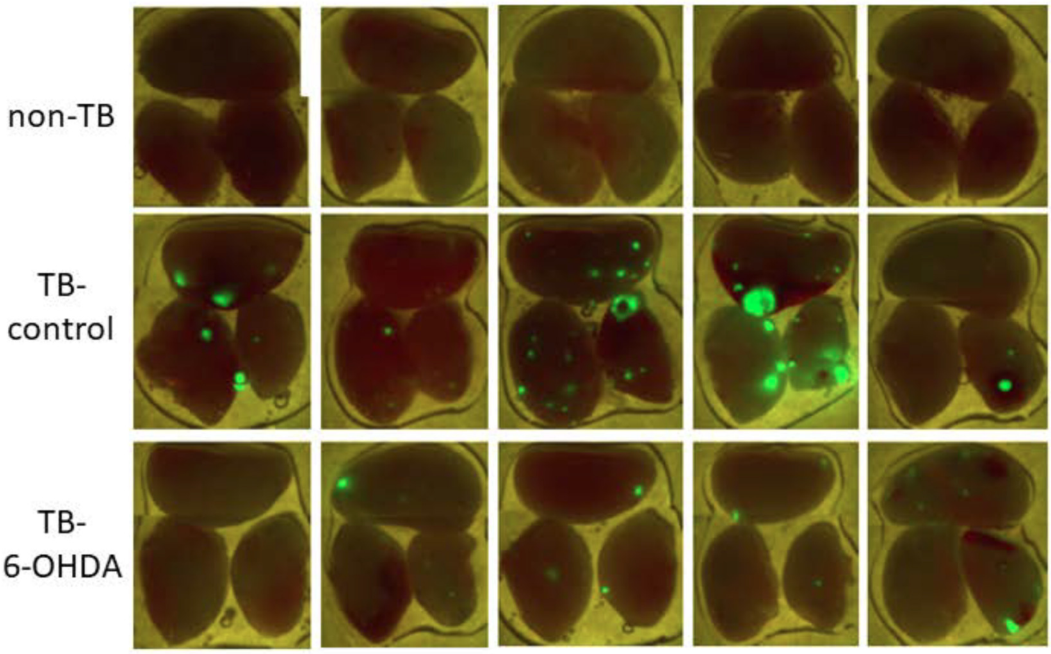

Supplementary Figure S1. Decreased lung metastasis in sympathectomized lungs. Metastatic foci were observed by fluorescence stereoscopic microscope. Green dots showed metastatic foci.
